# Sevoflurane induces neuronal activation and behavioral hyperactivity in young mice

**DOI:** 10.1038/s41598-020-66959-x

**Published:** 2020-07-08

**Authors:** Lei Yang, Hoai Ton, Ruohe Zhao, Erez Geron, Mengzhu Li, Yuanlin Dong, Yiying Zhang, Buwei Yu, Guang Yang, Zhongcong Xie

**Affiliations:** 1 Department of Anesthesiology, Ruijin Hospital, Shanghai Jiao Tong University School of Medicine, 200025 Shanghai, P. R. China; 2 Department of Anesthesia, Critical Care and Pain Medicine; Massachusetts General Hospital and Harvard Medical School, Charlestown, MA 02129-2060 USA; 3 Skirball Institute, Department of Neuroscience and Physiology, New York University School of Medicine, New York, NY 10016 USA; 4 Department of Anesthesiology, Shanghai First Maternity and Infant Hospital, Tongji University School of Medicine, Shanghai, P. R. China; 5 Department of Anesthesiology, Columbia University Irving Medical Center, New York, NY 10032 USA

**Keywords:** Neuroscience, Neurology

## Abstract

Sevoflurane, a commonly used anesthetic, may cause agitation in patients. However, the mechanism underlying this clinical observation remains largely unknown. We thus assessed the effects of sevoflurane on neuronal activation and behaviors in mice. Ten-day-old mice received 2% sevoflurane, 1% isoflurane, or 6% desflurane for 10 minutes. The behavioral activities were recorded and evaluated at one minute after the loss of righting reflex in the mice, which was about two minutes after the anesthetic administration. The neuronal activation was evaluated by c-Fos expression and calcium imaging at one minute after the anesthetic administration. Propofol, which reduces neuronal activation, was used to determine the cause-and-effect of sevoflurane. We found that sevoflurane caused an increase in neuronal activation in primary somatosensory cortex of young mice and behavioral hyperactivity in the mice at one minute after the loss of righting reflex. Desflurane did not induce behavioral hyperactivity and isoflurane only caused behavioral hyperactivity with borderline significance. Finally, propofol attenuated the sevoflurane-induced increase in neuronal activation and behavioral hyperactivity in young mice. These results demonstrate an unexpected sevoflurane-induced increase in neuronal activation and behavioral hyperactivity in young mice. These findings suggest the potential mechanisms underlying the sevoflurane-induced agitation and will promote future studies to further determine whether anesthetics can induce behavioral hyperactivity via increasing neuronal activation.

## Introduction

Each year, there are about 314 million patients who have surgery under anesthesia in the world^1^. Sevoflurane is one of the most commonly used inhalational anesthetics in children, and clinical investigations specifically showed that sevoflurane anesthesia was associated with the major epileptoid signs in children^2^. A clinical study investigating the effects of different concentrations of sevoflurane and propofol on electroencephalogram (EEG) in healthy people found that epileptiform discharges occurred in all eight participants at 1.5 and 2 MAC levels of sevoflurane anesthesia. Three participants showed electrographic seizures at 2 MAC level of sevoflurane, and propofol did not produce remarkable epileptiform EEG phenomena at any levels of anesthesia in the participants^3^.

However, the mechanism underlying this clinical observation of anesthesia-induced agitation remains unclear. Therefore, we set out to determine the effects of different anesthetics (sevoflurane, isoflurane, and desflurane) on neuronal activation and behavioral activity in young mice. The outcomes of the study would demonstrate that inhalational anesthetics may induce increases in neuronal activation and behavioral hyperactivity in mice, and will establish a system to determine the underlying mechanism by which patients develop agitation in the induction of general anesthesia or emergence from general anesthesia. These efforts may lead to understanding the underlying mechanism related to postoperative delirium observed in patients.

Despite the inherent limitations in all animal models, the mouse is the most common animal model for preclinical anesthesia studies because it is cost-efficient while containing a genome similar to the human genome (99%). In addition, the potency for the anesthetic-induced loss of righting reflex in mice correlates strongly with loss of perceptive awareness (consciousness) in humans for many anesthetics^4^. Moreover, the findings  that isoflurane, but not desflurane, induced cognitive dysfunction in mice have guided the clinically relevant pilot studies, which showed similar findings in humans^5,6^. Although we need to fully recognize the translational limitation, we expect the outcome from the current studies in mice to be translatable to humans. In particular, the increases in neuronal activation and behavioral hyperactivity in mice could recapitulate the observed agitation and underlying mechanisms in patients. However, these changes in mice may only partially explain the agitation and the underlying mechanism in patients and other underlying mechanisms may also contribute to the agitation in patients.

Hyperactive behavior in rodents is manifested by body movements such as increased locomotor activity, epileptic-like movements, and seizures^7^. Specifically, Lim and colleagues used a modified method, which was used initially to quantify seizure behavior^8^, to assess the behavior of rats and demonstrated the hyperactivity in young rats after the administration of sevoflurane^9^. Moreover, Liang and colleagues reported that sevoflurane was able to increase the locomotor activity in young, but not adult, mice during the first two minutes after the administration of 1% sevoflurane and the six minutes after the recovery from the anesthesia^10^. Finally, Ton and colleagues also showed an increase of locomotor activity in mice during the induction of sevoflurane anesthesia and this increase in locomotor activity lasted for five minutes after the recovery from the sevoflurane^11^. However, these studies did not assess the neuronal activity of the mice at the time when they exhibited hyperactive behavior. Therefore, the neuronal mechanisms underlying the anesthesia-associated behavioral hyperactivity remain unknown.

Expression of c-Fos has been used to indicate the increased neuronal activation induced by pain^12^, heroin-seeking behavior^13^, chronic stress^14^, post-traumatic stress order^15^, electric stimulation^16^, and schizophrenia^17^. Therefore, in the present study, we measured the expressions of c-Fos in mouse brain following the administration of sevoflurane to determine the effects of sevoflurane on neuronal activation.

Recently, with the development of genetically encoded calcium indicator GCaMP, the levels of intraneuronal calcium can be assessed by expressing GCaMP in specific cell types or subcellular compartments^18^. Thus, in the present studies, we used *in vivo* two-photon microscopy and genetically-encoded calcium indicator GCaMP6 slow (GCaMP6s) to perform calcium imaging in layer 2/3 (L2/3) neuronal somata in the mouse primary somatosensory cortex to measure the changes of neuronal activity following the administration of sevoflurane *in vivo*.

In the current studies, we assessed the effects of anesthetics on the behavior and neuronal activity in young mice. We used *in vivo* and *in vitro* approaches to test a hypothesis that sevoflurane increases neuronal activation, which is associated with behavioral hyperactivity in young mice.

Propofol, a commonly used intravenous anesthetic^19^, primarily potentiates GABAA receptor activity^20^, enhances GABA signaling^21–23^, and inhibits glutamatergic activation in hippocampal neurons^24^, leading to reduced neuronal activation^25–27^. We, therefore, used propofol to determine the potential cause-and-effect relationship of the increased neuronal activation and behavioral hyperactivity following the administration of sevoflurane in the young mice.

## Results

### Sevoflurane induced behavioral hyperactivity in young mice

**Figure 1:**
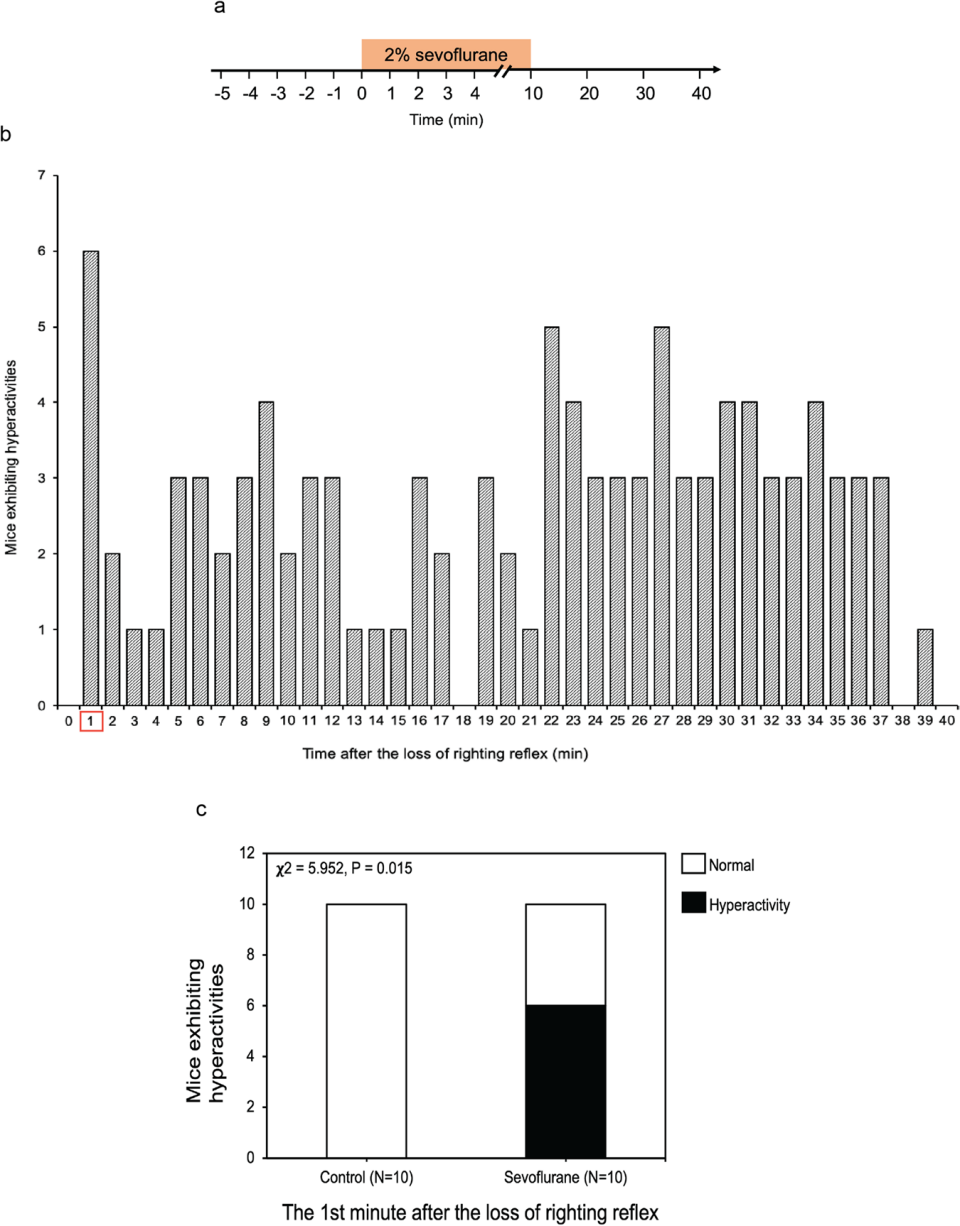
Sevoflurane induced behavioral hyperactivity in young mice. (**a**) Timeline of the experiment. The activity of each of P10 mice was recorded before, during, and after the administration of 2% anesthetic sevoflurane for 10 minutes (indicated by the box). **(b)** The number of mice exhibiting hyperactivity after the loss of righting reflex. **(c)** At the first minute after the loss of righting reflex (induced by the administration of 2% sevoflurane), 60% of the young mice exhibited hyperactivity (**χ**^2^ = 5.952, *P* = 0.015, *n* = 10, **χ**^2^ test).

We first established the system by treating the postnatal 10 (P10) mice with 2% sevoflurane for 10 minutes and then observing the behavior of the mice up to 40 minutes (Fig. 1a). There was an average of one minute between the time of administration of sevoflurane and the time of loss of righting reflex (data not shown). We found that the administration of 2% sevoflurane induced a behavioral hyperactivity in P10 mice at the first minute after the loss of righting reflex, which was about two minutes after the sevoflurane administration (Fig. 1b and the video in Supplemental Data), with 60% of mice (*n* = 10) exhibiting hyperactivity (Fig. 1c; **χ**^2^ = 5.952, *P* = 0.015). These data indicated that sevoflurane induced behavioral hyperactivity in young mice.

We also found that 40% of P10 mice (*n* = 10) exhibited hyperactivity (Supplementary Fig. S1c; **χ**^2^ = 5.000, *P* = 0.087) at the 9^th^ minute after the recovery of righting reflex (Supplementary Fig. S1). Therefore, we focused on the first minute after the loss of righting reflex in mice in the following experiments.

**Figure 2:**
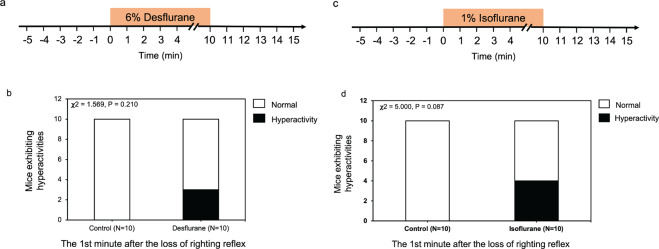
Isoflurane and desflurane did not induce behavioral hyperactivity in young mice. (**a**) Timeline of the experiment. The activity of each of P10 mice was recorded before, during, and after the administration of 6% desflurane for 10 minutes (indicated by the box). **(b)** At the first minute after the loss of righting reflex, 30% of the young mice (*n* = 10) exhibited hyperactivity in the 6% desflurane group (**χ**^2^ = 1.569, *P* = 0.210). **(c)** Timeline of the experiment. The activity of each of P10 mice was recorded before, during, and after the administration of 1% isoflurane for 10 minutes (indicated by the box). **(d)** At the first minute after the loss of righting reflex, 40% of the young mice (*n* = 10) exhibited hyperactivity in the 1% isoflurane group (**χ**^2^ = 5.000, *P* = 0.087).

A group of P10 mice were exposed to 6% desflurane, another inhalation anesthetic with a stronger pungent smell than sevoflurane^28^ for 10 minutes (Fig. 2a). 6% desflurane did not induce significant behavioral hyperactivity in P10 mice (Fig. 2b**; χ**^2^ = 1.569, *P* = 0.210) at the first minute after the loss of righting reflex as compared with the control condition. Another group of mice were exposed to 1% isoflurane, another commonly used inhalational anesthetic for 10 minutes (Fig. 2c). And 1% isoflurane only induced behavioral hyperactivity with borderline significance in P10 mice (Fig. 2d**; χ**^2^ = 5.000, *P* = 0.087). Therefore, we focused on the sevoflurane in the following mechanistic experiments.

### Sevoflurane increased the number of c-Fos-positive cells in the somatosensory cortex of young mice

**Figure 3:**
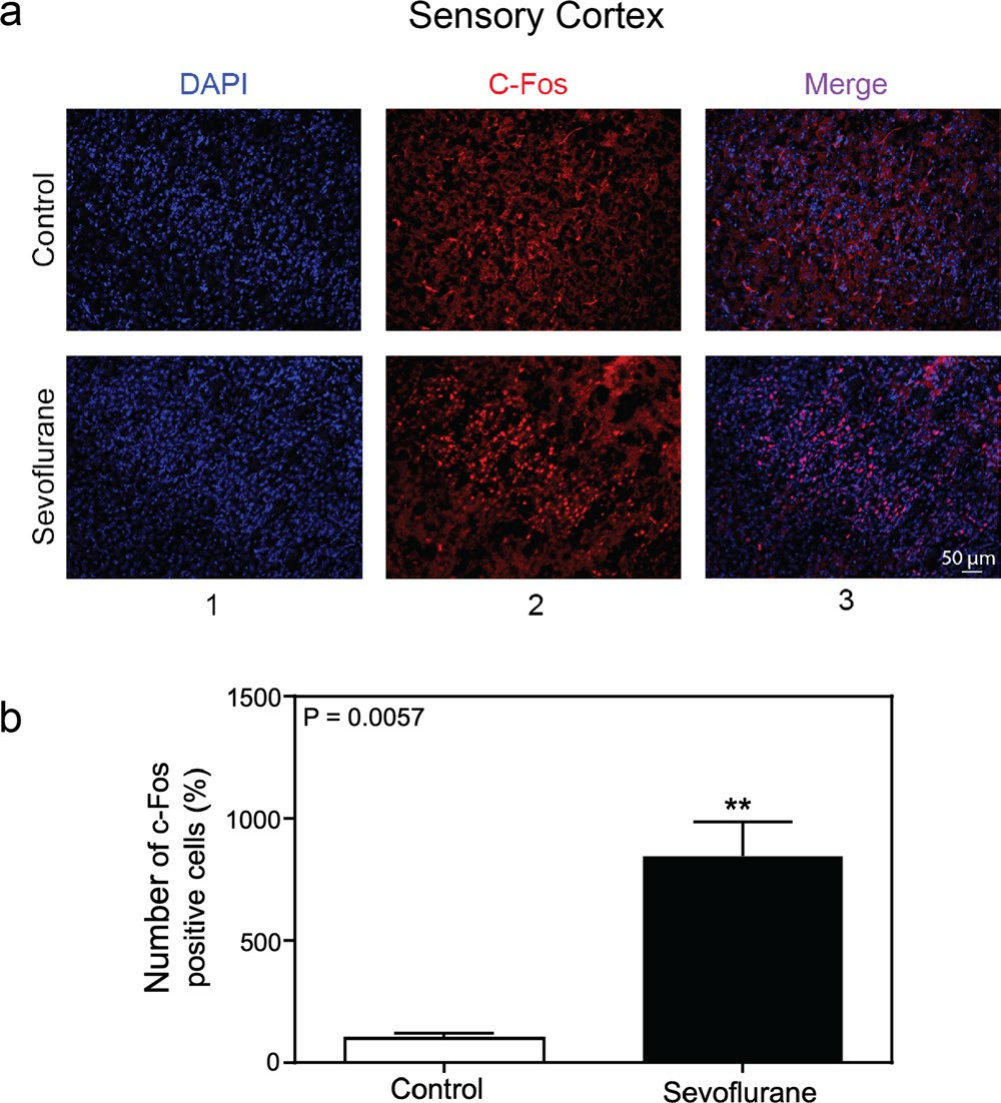
Sevoflurane increased the number of c-Fos-positive cells in the somatosensory cortex of young mice. (**a**) Immunohistochemistry staining of c-Fos in sensory cortex (20×). Column 1 is the image of nuclei (blue), column 2 is the image of c-Fos (red), and column 3 is the merged image. The top row represents the brain tissues of mice following the control condition, and the bottom row represents the brain tissues of mice treated with 2% sevoflurane (20 ×). **(b)** Quantification of the immunohistochemistry staining showed that the anesthetic sevoflurane increased the number of the c-Fos-positive cells as compared to the control condition in the sensory cortex of the mice (847.8% versus 100%, *P* = 0.0057, *n* = 3, *t* test).

To search for the effects of anesthetics on neuronal activity related to sevoflurane-induced behavioral hyperactivity during anesthesia, we performed c-Fos staining in the entire brain harvested at the first minute after the lost of righting reflex in P10 mice. As compared to the control condition, there were increases in c-Fos positive neurons in the primary sensory cortex, basolateral amygdala and ventromedial hypothalamus following the sevoflurane administration (data not shown). Moreover, we observed a more robust increase in c-Fos positive neurons in primary somatosensory cortex following the sevoflurane administration as compared to the control condition (Fig. 3a). The quantification of immunohistochemistry images showed increases in c-Fos-positive cells following the administration of sevoflurane as compared to the control condition: 847.8% versus 100%, *P* = 0.0057, *n *= 3 (Fig. 3b). These data suggest that the administration of sevoflurane can induce neuronal activation in the primary somatosensory cortex of the young mice.

### The behavioral hyperactivity and neuronal activation in young mice were not induced by pungent smell

To test whether the behavioral hyperactivity and neuronal activation observed in these mice was due to the pungent smelling of sevoflurane, we assessed the number of c-Fos-positive cells in the olfactory bulb of young mice after the administration of sevoflurane. We found that sevoflurane did not increase the number of c-Fos-positive cells in the olfactory bulb of the young mice (Supplementary Fig. S2). These data suggest that the behavioral hyperactivity and neuronal activation observed in the young mice following the administration of sevoflurane was unlikely due to the pungent smelling of sevoflurane.

### Sevoflurane increased activity of cortical pyramidal neurons during anesthesia induction

**Figure 4:**
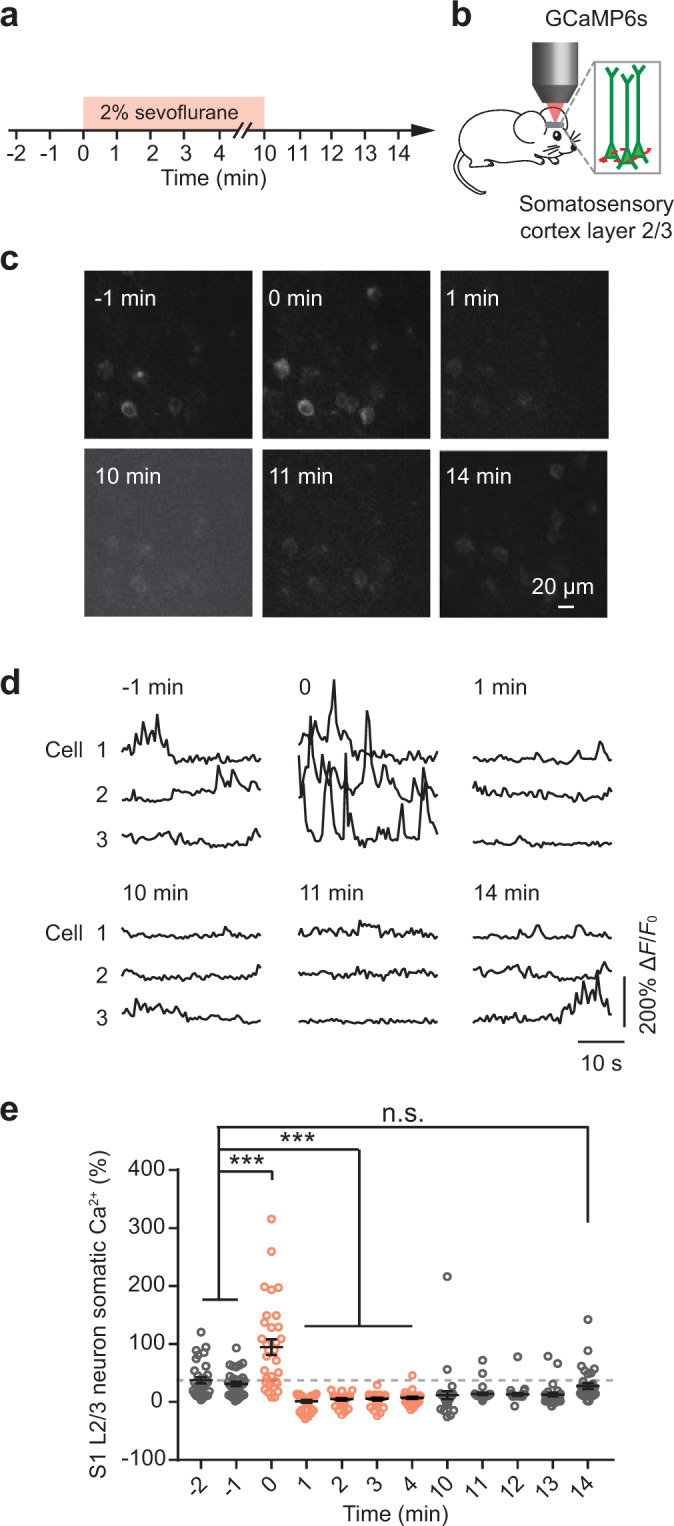
Sevoflurane increased neuronal activity in the primary somatosensory cortex of young mice. (**a**) Timeline of the experiment. The neuronal activity in P10 mice was recorded before, during, and after the administration of 2% sevoflurane for 10 minutes (indicated by the orange box). (**b**) Schematic diagram showing *in vivo* two-photon imaging in layer 2/3 (L2/3) of the primary somatosensory cortex (S1). (**c**) Representative images of L2/3 somata in S1 expressing GCaMP6s at the indicated time points. Scale bar: 20 µm. (**d**) Representative calcium fluorescence traces from 3 cells in one mouse at the indicated time points. (**e**) Summary quantification of the neuronal calcium activity averaged over 30 seconds at each time point (-2 minute: 37.7 ± 5.4%; −1 minute: 31.4 ± 3.9%; 0 minute: 94.7 ± 14.5%; 1 minute: 1.1 ± 2.3%; 2 minute: 5.2 ± 1.9%; 3 minute: 5.9 ± 1.9%; 4 minute: 8.3 ± 1.8%; 10 minute: 11.9 ± 7.1%; 11 minute: 14.1 ± 2.3%; 12 minute: 13.3 ± 2.2%; 13 minute: 12.8 ± 3.1%; 14 minute: 27.7 ± 5.2%, 32 cells from 4 mice; ***P < 0.001, n.s. *P* > 0.05, one-way ANOVA). Summary data are presented as mean ± s.e.m. Each circle represents an individual cell.

To further understand the sevoflurane-induced neuronal activation in the primary somatosensory cortex, we performed *in vivo* calcium imaging in layer 2/3 (L2/3) pyramidal neurons expressing the genetically-encoded calcium indicator GCaMP6s. At P10, mice were administered 2% sevoflurane for 10 minutes (Fig. 4a). The two-photon calcium imaging was performed before (−2 to 0 minute), during (0 to 10 minute), and after (10 to 14 minute) the administration of sevoflurane (Fig. 4b,c). We found that the calcium levels in the somas of L2/3 neurons were about 3-fold higher within the first minute of sevoflurane administration as compared to that during pre-administration awake state (Fig. 4d,e; total integrated Δ*F*/*F*_0_: 94.7 ± 14.5% at 0 minute vs. 31.4 ± 3.9% at −1 minute, 32 cells from 4 mice, *P* < 0.001). One to two minutes after the administration of sevoflurane, the activity of cortical neurons was significantly lower than that during awake state (total integrated Δ*F*/*F*_0_: 1.1 ± 2.3% at 1 minute vs. 31.4 ± 3.9% at −1 minute, 32 cells from 4 mice, *P* < 0.001). This reduced neuronal activity persisted through the rest of the anesthesia period (Fig. 4d,e). Upon withdrawal of sevoflurane, the activity of cortical neurons recovered to their pre-administration baseline level within 5 minutes (Fig. 4e; total integrated Δ*F*/*F*_0_: 27.7 ± 5.2% at 14 minute vs. 31.4 ± 3.9% at −1 minute; 32 cells from 4 mice, *P* > 0.05). Taken together, these data revealed a transient elevation of neuronal activity after sevoflurane administration in young mice, which could be related to the observed behavioral hyperactivity during anesthesia induction.

### Propofol attenuated the sevoflurane-induced behavioral hyperactivity and neuronal activation in young mice

**Figure 5:**
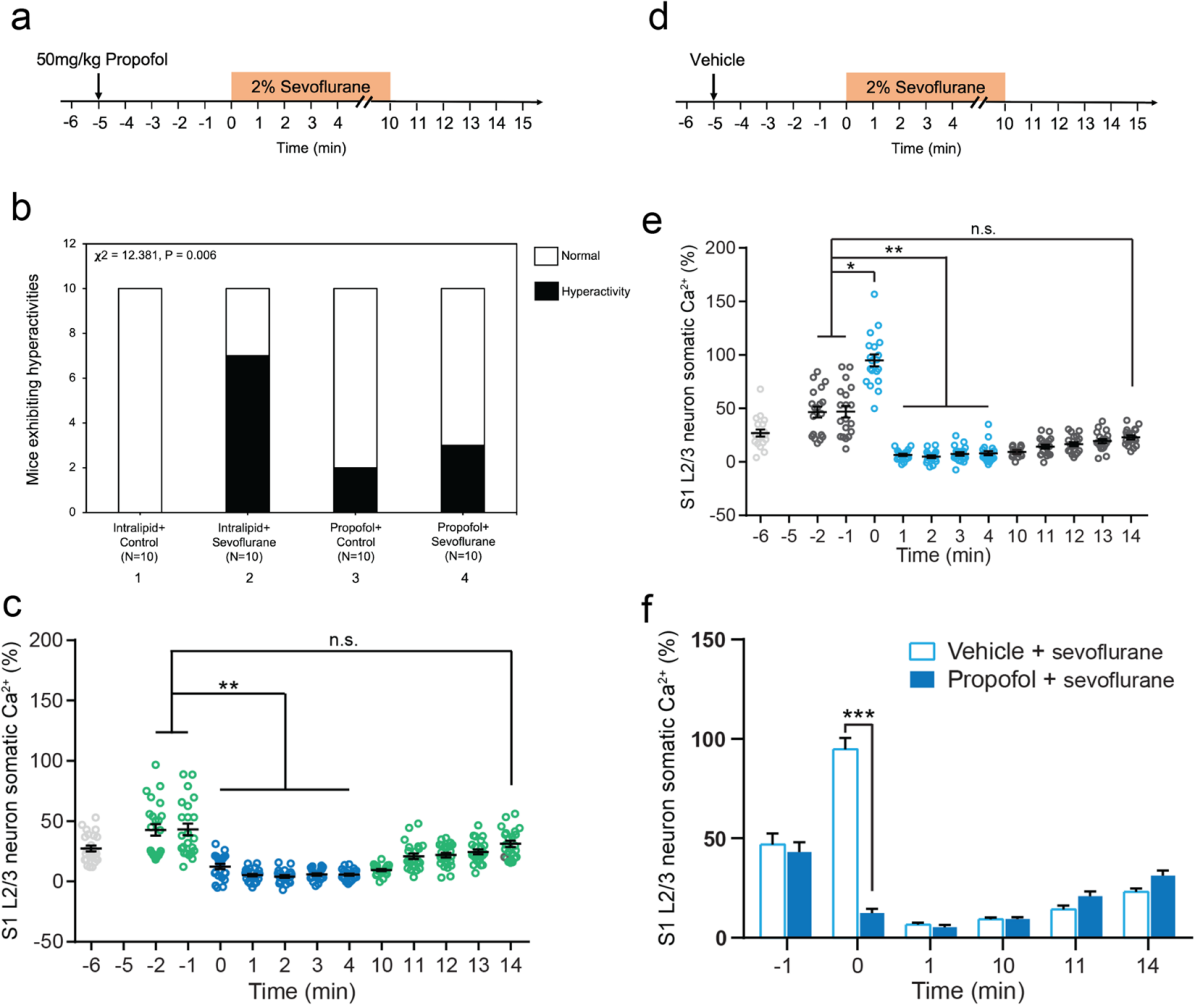
Propofol attenuated the sevoflurane-induced behavioral and neuronal hyperactivity in young mice. (**a**) Timeline of the experiment. 50 mg/kg propofol was injected intraperitoneally 5 minutes before the administration of 2% sevoflurane. Mice were exposed to 2% sevoflurane for 10 minutes. Afterward, mice were left in the chamber to recover for 5 minutes. The behavior of each mouse was recorded for 20 minutes. **(b)** At the first minute after the administration of 2% sevoflurane plus intralipid (the vehicle of propofol), 70% of young mice exhibited hyperactivity (bar 2). 20% of young mice exhibited hyperactivity after the administration of intravenous anesthetic propofol (bar 3). Finally, 30% of young mice exhibited hyperactivity following the administration of propofol plus sevoflurane (bar 4) (**χ**^2^ = 12.381, *P* = 0.006, *n* = 10, **χ**^2^ test). These data demonstrated that propofol attenuated the sevoflurane-induced hyperactivity in young mice. (**c**) *In vivo* calcium imaging was performed in the S1 of P10 mice that received an intraperitoneal injection of propofol (at −5 minute) and then sevoflurane (at 0 minute). Light grey is baseline without any treatment. Green is propofol only. Dark blue is propofol + sevoflurane. (**d**) Timeline of vehicle (intralipid) experiment. *In vivo* calcium imaging was performed in the S1 of P10 mice that received an intraperitoneal injection of intralipid (vehicle of propofol, indicated by the arrow, at −5 minute). Five minutes after the administration of the vehicle, the mice received the administration of 2% sevoflurane (at 0 minute). (**e**) Summary quantification of neuronal calcium activity averaged over 30 seconds at each time point. Light grey is baseline without any treatment. Dark grey (−2 minute, −1 minute) is vehicle. Light blue is vehicle + sevoflurane. (**f**) Comparison of neuronal calcium activity in P10 mice between propofol plus sevoflurane treatment and vehicle plus sevoflurane treatment (*t*-test). Summary data are presented as mean ± s.e.m. Each circle represents an individual cell. *P < 0.05, **P < 0.01, ***P < 0.001, n.s. *P* > 0.05.

Propofol, a commonly used intravenous anesthetic, could inhibit neuronal activation in hippocampal neurons^24^. We then tested whether propofol could attenuate the sevoflurane-induced behavioral hyperactivity and neuronal activation in young mice. 50 mg/kg propofol (Fig. 5a) or vehicle (intralipid, Fig. 5d) was administrated to P10 mice 5 minutes before the administration of sevoflurane. We found that whereas sevoflurane still induced behavioral hyperactivity in the mice pretreated with vehicle, sevoflurane did not cause behavioral hyperactivity in the mice pretreated with propofol (Fig. 5b; **χ**^2^ = 12.381, *P* = 0.006, *n* = 10).

Similarly, sevoflurane did not increase neuronal activity in mice pretreated with propofol (Fig. 5c), yet still increased neuronal activity in mice pretreated with vehicle (Fig. 5d,e). Finally, the summary quantification of the neuronal activity illustrated that propofol attenuated the sevoflurane-induced neuronal activation in P10 mice within the first minute after the administration of sevoflurane (Fig. 5f). These data linked anesthesia-induced neuronal activation in the somatosensory cortex with the behavioral hyperactivity in young mice.

Interestingly, the administration of propofol in P10 mice (at −5 minute, Fig. 5a) caused slight neuronal activation but prevented the sevoflurane-induced further neuronal activation in the young mice (Fig. 5c; total integrated Δ*F*/*F*_0_: −6 minute: 33.3 ± 4.0%; −2 minute: 42.8 ± 4.8%; −1 minute: 43.2 ± 4.8%; 0 minute: 12.5 ± 2.1%, 1 minute: 5.3 ± 1.1%; 2 minute: 4.1 ± 1.1%; 3 minute: 5.9 ± 1.0%; 4 minute: 5.7 ± 0.9%, 23 cells from 3 mice). It is possible that the initial elevation of neuronal activity prevented the further elevation of neuronal activity after the administration of sevoflurane, a potential pre-conditioning mechanism. Future research to test this hypothesis is warranted. Propofol caused a moderate elevation of pyramidal neuron activity in the somatosensory cortex of P10 mice without causing the behavioral hyperactivity (Fig. 5b,c,f). These data suggest that mice may not exhibit behavioral hyperactivity with only moderate neuronal activation in the cortex. Future studies to test this hypothesis are also warranted.

### Propofol attenuated the sevoflurane-induced increase in the number of c-Fos-positive cells in the somatosensory cortex of young mice

**Figure 6:**
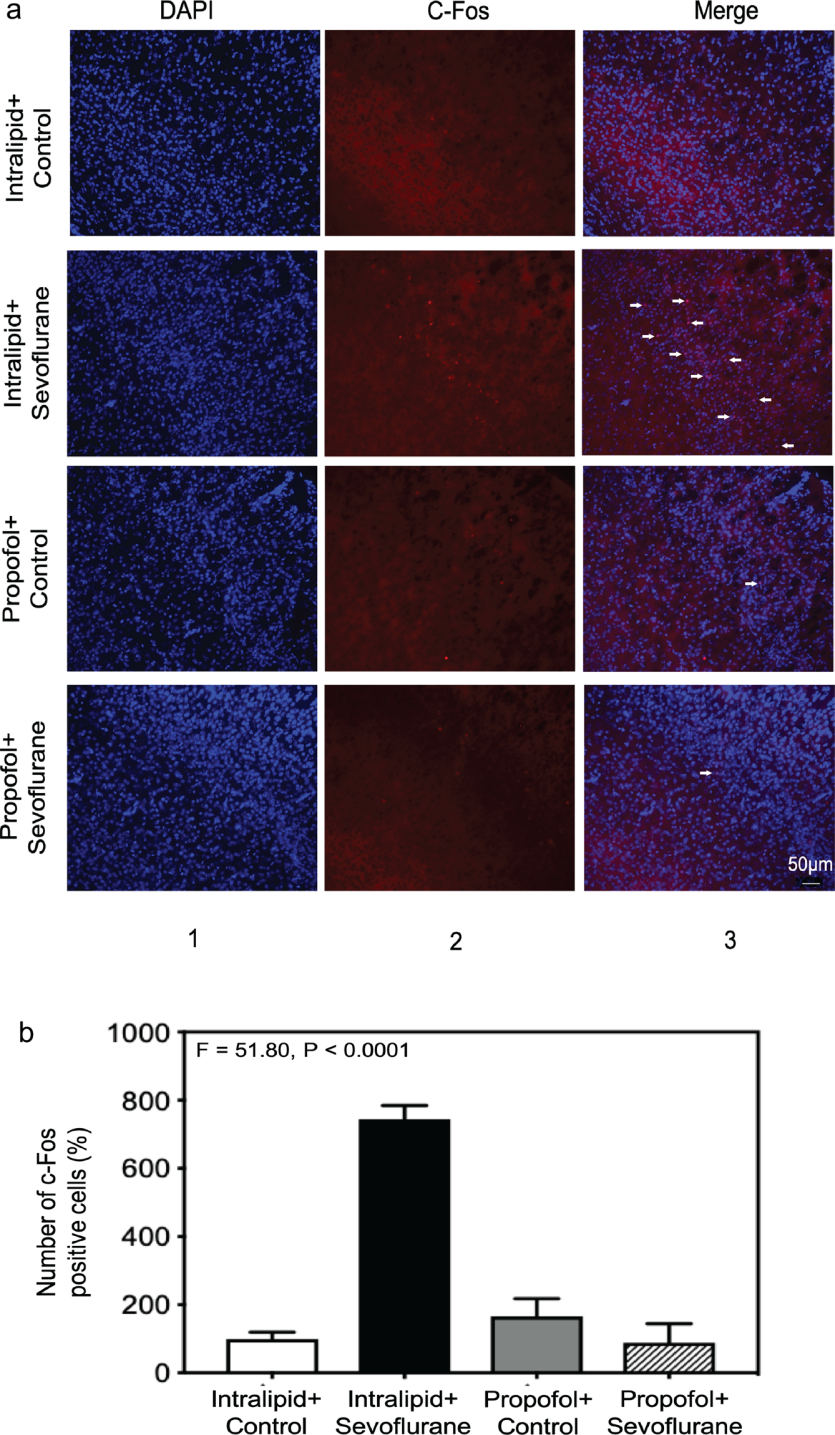
Propofol attenuated the sevoflurane-induced increase in the number of c-Fos-positive cells in the somatosensory cortex of young mice. (**a**) Immunohistochemistry staining of c-Fos (magnification 20 ×). Column 1 is the image of nuclei (blue), column 2 is the image of c-Fos (red), and column 3 is the merged image. Top row represents the brain tissues of mice following the intralipid plus control condition, the second row represents the brain tissues of mice treated with intralipid plus 2% sevoflurane, the third row represents the brain tissues of mice treated with 50 mg/kg propofol, and the fourth row represents the brain tissues of mice treated with 50 mg/kg propofol plus 2% sevoflurane (20 ×). **(b)** Quantification of the immunohistochemistry staining showed that sevoflurane increased the number of c-Fos-positive cells as compared to the control condition. Propofol attenuated the sevoflurane-induced increase in the number of c-Fos-positive cells in the somatosensory cortex of the young mice (*F* = 51.80, *P* < 0.0001, *n* = 3, one-way ANOVA).

Consistent with the results in calcium imaging studies, immunohistochemistry staining of c-Fos after the administration of propofol or intralipid showed that propofol decreased the c-Fos-positive cells in the primary somatosensory cortex at first minute after sevoflurane administration (Fig. 6a). Sevoflurane plus intralipid (row 2) increased the number of c-Fos-positive cells as compared with control plus intralipid condition (row 1). However, propofol attenuated the sevoflurane-induced increase in the number of c-Fos-positive cells (row 4) (Fig. 6b; *F* = 51.80, *P* < 0.0001, *n* = 3). These data further suggest that propofol can attenuate the sevoflurane-induced neuronal activity in young mice.

### Sevoflurane increased the intracellular calcium levels in cultured neurons

We established an *in vitro* calcium imaging system to determine the effects of sevoflurane, at different concentrations, on intracellular calcium levels in cultured neurons. Calcium imaging showed that treatment with 0.45 mM (2%) sevoflurane increased intracellular calcium levels right after we applied the sevoflurane and decreased them after we started applying the bath solution in cultured neurons, whereas treatment with 0.11 mM (0.5%) and 0.22 mM (1%) sevoflurane did not increase the intracellular calcium levels in the neurons. Quantification of calcium imaging, based on the ratio of Δ*F* to *F*_0_, revealed that 0.45 mM (2%) sevoflurane increased the intracellular calcium levels as compared to control condition in the neurons: 0.04992 (fluorescent intensity, F) versus 0.02721 (fluorescent intensity, F), *P* < 0.05 (Supplementary Fig. S3).

### Propofol attenuated the sevoflurane-induced increase in intracellular calcium levels in primary neurons

**Figure 7:**
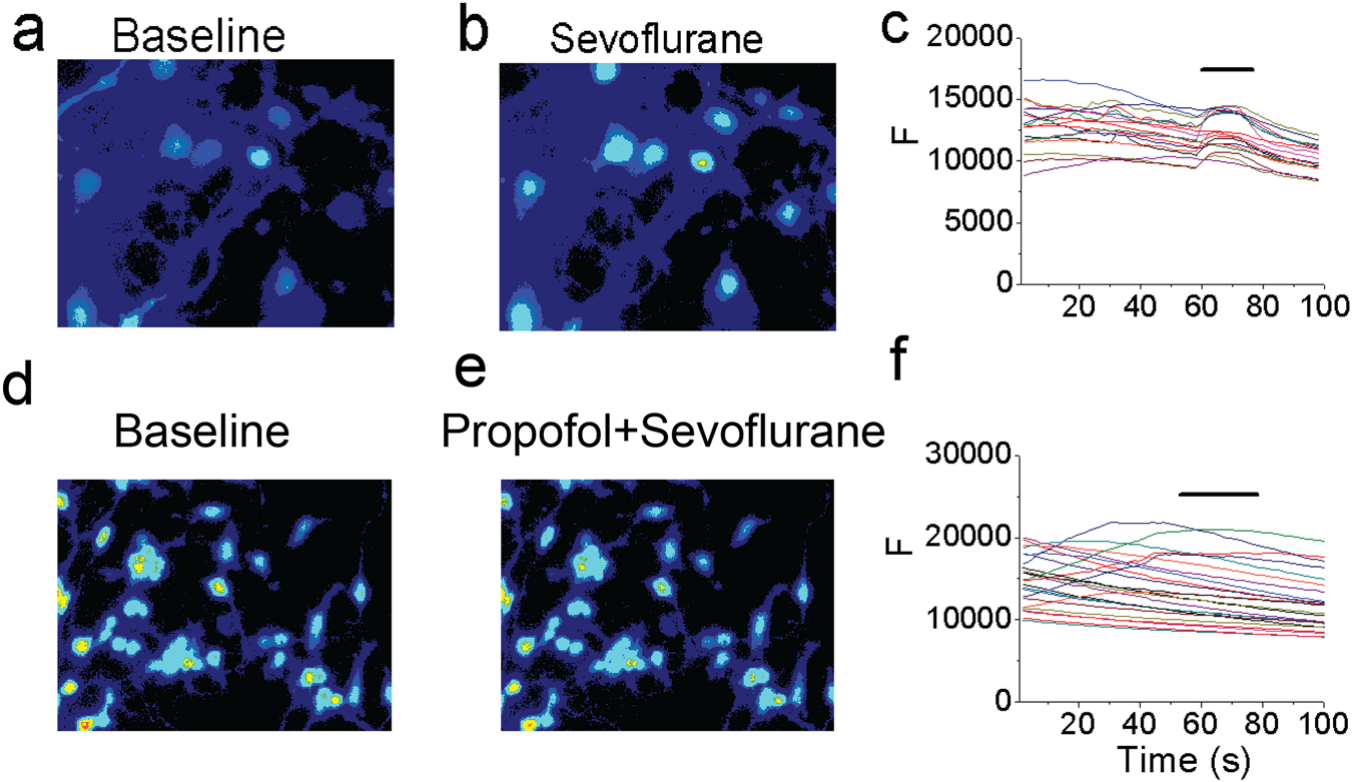
Propofol attenuated the sevoflurane-induced increase in intracellular calcium levels in primary neurons. (**a**) The baseline intracellular calcium imaging in the neurons. **(b)** The intracellular calcium imaging in the neurons treated by 0.45 mM (2%) sevoflurane. (**c**) The quantification of the intracellular calcium imaging of a and b. (**d**) The baseline intracellular calcium imaging in the neurons. **(e)** The intracellular calcium imaging in the neurons treated by 0.45 mM (2%) sevoflurane plus propofol (10 µM). (**f**) The quantification of the intracellular calcium imaging of d and e. F means fluorescence intensity.

To understand the mechanisms underlying sevoflurane-induced neuronal activation, we assessed the effects of sevoflurane on somatic calcium levels in primary neurons, and the effects of propofol. KCl, the positive control in enhancing the intracellular calcium levels^29^, increased the intracellular calcium levels in the primary neurons (Supplementary Fig. S4). The peak amplitude of KCl-induced response was inhibited if we pretreated the cell with propofol (see KCl-induced response in Supplementary Fig. S4c,g). Using this established *in vitro* calcium imaging system, we found that administration of 2% sevoflurane increased intraneuronal calcium levels (Fig. 7a,b,c). Propofol attenuated the sevoflurane-induced elevation of intraneuronal calcium levels (Fig. 7d,e,f). These data support the hypothesis that sevoflurane might induce neuronal activation and propofol was able to attenuate the sevoflurane-induced neuronal activation in primary cultured neurons, consistent with the observation in young mice.

## Discussion

Unexpectedly, we found that inhalational anesthetic sevoflurane was able to increase neuronal activation in somatosensory cortex of young mice (P10) within one minute after the administration of sevoflurane, and also to induce behavioral hyperactivity at one minute after the loss of righting reflex, which was about two minutes after the administration of sevoflurane. Moreover, intravenous anesthetic propofol attenuated the sevoflurane-induced neuronal activation and behavioral hyperactivity in young mice. These findings suggest that general anesthesia can induce the  activation of somatosensory cortex neurons, leading to hyperactivity rather than sedation of behavior in young mice. These findings provide further evidence that elements of increased neuronal activity and hyperactive behavior can occur during the general anesthesia state. The findings that the propofol treatment attenuated the sevoflurane-induced neuronal activation and behavioral hyperactivity suggest the cause-and-effect relationship between the neuronal activation and behavioral hyperactivity during the sevoflurane anesthesia in young mice. However, such conclusion may still need to be further investigated using the system established in the current study.

Importantly, the findings that inhalational anesthetic sevoflurane can cause neuronal activation and induce behavioral hyperactivity in mice suggest that the anesthetic-induced neuronal activation can contribute, at least partially, to the observed agitation during the induction of general anesthesia or emergence from general anesthesia in patients. These results may further help us to determine the underlying mechanism related to postoperative delirium observed in patients, leading to development of strategies to prevent or treat postoperative delirium, which is associated with a high risk of developing Alzheimer’s Disease and related dementias, greater incidences of postoperative complications, higher mortality, prolonged hospital stays, and higher discharge rates to nursing homes^30–34^.

Expression of c-Fos serves as a marker for neuronal activity^35–37^. Under basal conditions, the c-Fos protein maintains a low level of expression since the mRNA and protein of c-Fos can inhibit its generation^38^. Because c-Fos is often expressed when neurons fire action potentials, upregulation of c-Fos levels in neurons indicates the neuronal activation. Specifically, several studies have shown that pain^12^, heroin-seeking behavior^13^, chronic stress^14^, post-traumatic stress order^15^, electric stimulation^16^, schizophrenia^17^ can induce neuronal activation, which is indicated by the higher expression of c-Fos. In the present study, we measured the levels of c-Fos in different regions of the mouse brain, including the primary sensory cortex, following the administration of sevoflurane to determine the effects of sevoflurane on neuronal activation.

Propofol is a commonly used intravenous anesthetic^19^ and can enhance GABA neurotransmission and inhibit glutamatergic neurotransmission. Specifically, propofol has been shown to enhance GABA release from presynaptic cortical nerve terminals^39^ and to increase GABA-evoked chloride currents in cultured cortical neurons^40^ and brain slices^41^. Kitamura *et al*. also showed that propofol could increase the open probability of postsynaptic GABA receptors by reducing the slow phase of closed receptors^42^. In addition, propofol regulates the intrinsic membrane properties by modulating the extra-synaptic GABA receptor^43^. On the other hand, propofol inhibits presynaptic sodium channels, resulting in the reduction of glutamatergic synaptic transmission^44^, normalizes the increased expression of glutamate in the hippocampus of rats with sleep deprivation^45^, and inhibits the veratridine-evoked glutamate release in the synaptosomes from rat brain regions^46^. Finally, propofol can reverse the oxygen-glucose deprivation-induced elevation of the extracellular glutamate concentrations by reducing glutamate uptake^47^, and inhibits glutamatergic activation by acting on protein kinases^24^. We, therefore, used propofol as a tool to determine whether propofol can attenuate both neuronal activation and behavioral hyperactivity in the mice anesthetized by sevoflurane, which could demonstrate the cause-and-effect relationship of the neuronal activation and behavioral hyperactivity in mice. The findings that propofol could attenuate the neuronal activation and behavioral hyperactivity induced by sevoflurane support the cause-and-effect relationship of neuronal activation and behavioral hyperactivity. However, sevoflurane could also induce the behavioral hyperactivity via different mechanism other than the increased neuronal activation. We will further investigate the mechanisms by which sevoflurane causes behavioral hyperactivity in the future studies, using the established system.

Sevoflurane-associated hyperactive behavior has been reported previously. Specifically, Lim and colleagues showed that anesthesia with 6% sevoflurane for induction and 3% sevoflurane for maintenance for 9 minutes caused general hyperactivity between 2 and 15 minutes after termination of anesthetic administration^9^. This study, however, did not specifically identify the timing when the mice demonstrated the hyperactivity. Liang and colleagues showed that the administration of 1% sevoflurane for two minutes, 2% sevoflurane for two minutes and then 3% sevoflurane for two minutes in both young and adult mice caused increases in the total locomotor activities in the period of 12 minutes after the administration of the anesthesia^10^. However, the temporal distribution of the animals’ locomotor activity after the anesthesia was not studied. Our previous study demonstrated that administration of sevoflurane increased the locomotion, as evidenced by the increases in total walking distance and velocity of movement, in adult mice during the induction and recovery period of the anesthesia^11^.

However, all of these studies did not assess the neuronal activation in the animals. Here, we performed two-photon calcium imaging in awake mice to determine the neuronal activation in the mouse somatosensory cortex. We found that the neurons exhibited a sudden and brief (less than 1 minute) increase (3-fold versus awake status) in intracellular calcium levels upon the administration of sevoflurane. With the continuous administration of sevoflurane, the activation of these neurons quickly decreased to the level that was lower than that under the awake condition and recovered to the pre-anesthesia baseline level after the stop of sevoflurane administration. The results indicated that sevoflurane briefly and reversibly increased the neuronal activation in the somatosensory cortex of living mice.

The anesthesia-associated agitation occurs during both the  induction of the anesthesia or the emergence of the anesthesia (reviewed in^48^). Specifically, it has been reported that the sevoflurane-induced hyperactivity and excitation are common among patients during the induction of anesthesia in the form of body movement, epileptiform EEG, epileptic movement, and even seizures^49^. Consistently, we demonstrated that the administration of sevoflurane caused brief (one minute after the loss of righting reflex) behavior hyperactivity in young mice. Taken together, it is possible that the administration of sevoflurane with specific concentrations (e.g., 2% sevoflurane) can increase the neuronal activation, leading to behavioral hyperactivity.

Our research showed that inhalational anesthetic isoflurane and desflurane did not induce significant behavioral hyperactivity in young mice. The reason why sevoflurane induced, but isoflurane or desflurane did not induce, the behavioral hyperactivity is not known at present. Our previous study also showed that sevoflurane, but not desflurane, caused neuroinflammation and cognitive impairment in young mice^50^. Notably, 1% isoflurane (1 MAC) induced a borderline behavioral hyperactivity in young mice (Fig. 2c,d). These findings suggest that isoflurane could induce a concentration-dependent behavioral hyperactivity in young mice, which is supported by the previous studies that 0.4–0.5 MAC isoflurane increased the spontaneous firing rate while 1.5 MAC isoflurane decreased it^51^.

Extracellular stimulation can promote the immediate transcription of the *c-Fos* gene^52^. Under normal conditions, c-Fos maintains a lower level of expression^53^. Increases in neuronal activation are associated with the increase in c-Fos levels^54^. Thus, c-Fos can serve as a marker for neuron activation. Immunofluorescence staining showed that the administration of sevoflurane immediately increased c-Fos levels in the somatosensory cortex, further suggesting that administration of sevoflurane was able to induce neuronal activation.

It has been widely accepted that intravenous anesthetic propofol decreases the neuronal activity in the cortex, induces the reversible loss of consciousness^55^ and prevents the sevoflurane-induced agitation in pediatric patients^56^. Consistently, in the present study, propofol significantly decreased the sevoflurane-induced neuronal and behavioral hyperactivity. These findings revealed the association between the sevoflurane-induced neuronal activation and the sevoflurane-induced behavioral hyperactivity in young mice, and further suggest that sevoflurane-induced neuronal activation contributes, at least partially, to the sevoflurane-induced behavioral hyperactivity in young mice.

The present study was limited by the current results because we can’t determine the causal relationship between increases in neuronal activity in the somatosensory cortex and behavioral hyperactivity.

In conclusion, we demonstrated that the administration of sevoflurane increased neuronal activation and induced behavior hyperactivity in young mice. Moreover, we found that intravenous anesthetic propofol attenuated the sevoflurane-induced increases in neuronal activation and behavioral hyperactivity *in vivo*. Collectively, these studies demonstrate that general anesthetics can possibly induce hyperactive behavior during the induction, maintenance, and recovery from general anesthesia. A general anesthetic-induced transient increase in neuronal activation in the somatosensory cortex may be involved in the mediation of such a behavioral phenotype. These efforts have generated a new concept and will promote further studies into anesthesia-associated agitation and other potential adverse effects, including postoperative delirium. These studies may ultimately improve the postoperative outcomes of the patients, especially pediatric patients.

## Methods

This experimental protocol was approved by the Massachusetts General Hospital Standing Committee on the Use of Animals in Research and Teaching (Protocol Number: 2006N000219) and the Institutional Animal Care and Use Committee at the Columbia University (Protocol Number: AAAW7462) and New York University (Protocol Number: 161116). All experiments were performed in accordance with relevant guidelines and regulations.

### Animal

Two or three-month-old C57BL female mice and male  mice, all purchased from the Jackson Laboratory (Bar Harbor, USA), were housed in the ventilated cage with free access to food and water. We used 10-day old mice in the current study. The temperature of the room was set at 24 ± 1 °C, and the humidity was controlled at 55% ± 5% with a 12-hour light/dark cycle (lights on at 7:00 am). The mice were handled following the rules of the NIH animal care and efforts were made to minimize the number of animals used as well as their suffering. The objective of the current study was not to investigate the sex-dependent effects; thus, we did not determine potential sex difference in the current studies. The mixture of male and female mice was used in the studies and randomly assigned into the different groups.

### Mice anesthesia

Mice at P10 in the anesthesia group received 2% sevoflurane plus 40% oxygen (balanced with nitrogen). A previous study found that arterial blood gas samples drawn from the spontaneously breathing rats at P4–P17 exposed to 2.1% sevoflurane showed no evidence of either hypoxia or hypoventilation^57^. Therefore, we choose 2% sevoflurane (1.0 MAC) as the treatment. The control conditions included oxygen (40% oxygen balanced with nitrogen) with an equal rate of flow in a chamber that was similar to that of the anesthesia chamber. The induction flow rate of fresh gas was 2 L/min from the start up to 2 minutes (for the induction of anesthesia) and then 1 L/min with the rest of the anesthesia (for the maintenance of anesthesia). The concentrations of anesthetics and oxygen were continuously monitored by using a gas analyzer (Dash 4000, GE Healthcare, USA) during the anesthesia administration. The anesthesia chamber temperature was monitored and controlled by a feedback-based system with the DC temperature control system (World Precision Instruments, USA), which monitors and automatically adjusts the temperature to keep the rectal temperature of each mouse at 37 °C (±0.5 °C) through a warming pad that is placed under the chamber. The mice were put in the chamber for 5 minutes to adapt to the environment and their behaviors were also recorded. The mice were then exposed to the anesthetics for 10 minutes. Afterward, the mice were left in the chamber to recover for 30 minutes. In the previous research of sevoflurane-induced hyperactivity behaviors, anesthesia in rats was induced with 6% (v/v) sevoflurane gas over 1 minute and then maintained with 3% sevoflurane gas for 9 minutes. Therefore, we used 10 minutes for the duration of the sevoflurane anesthesia^9^. The behavior of each mouse was recorded by a camera (F80, Samsung, Korea). Previous studies showed that 2.1% sevoflurane anesthesia did not significantly change the values of blood gas^57^. Thus, we did not determine the effects of 2% sevoflurane on the values of the blood gas in the present study. Another two groups of mice were exposed to 6% desflurane (1.0 MAC) and 1% isoflurane (1.0 MAC) for 10 minutes separately following the same protocol as that of the sevoflurane.

### Administration of propofol

50 mg/kg propofol or its vehicle (0.1 mL intralipid) was injected intraperitoneally 5 minutes before the administration of sevoflurane. Prevoius studies showed that  low dose propofol (50 mg/kg) did not induce the loss of righting reflex^58^. Therefore we gave mice 50 mg/kg propofol in the current study. Mice were then exposed to sevoflurane for 10 minutes. Afterward, mice were left in the chamber to recover for 5 minutes. The sevoflurane alone studies showed that the behavioral hyperactivity mainly occurred during the phase of induction of anesthesia. Thus, in the subsequent propofol plus sevoflurane studies, we only observed mice for a short period of time (5 minutes) during anesthesia recovery.

### Measure of loss of righting reflex (LORR)

Mice were put into a chamber and their behaviors were recorded. LORR was scored positive if the mouse remained on its back with at least three paws in the air for 60 seconds.  We determined that the average time from the administration of sevoflurane to the loss of righting reflex was 1 minute. This time was used as the time to the loss of righting reflex for each anesthetic.

### Quantification of the behavioral activity in mice

The behavioral activity in mice was scored using a method described in previous studies^9^. Specifically, each mouse was given a score between 0 to 6 on the basis of the following criteria: 0 – none; 1 – trembling; 2 – head bobbing or stereotypes; 3 – unilateral forelimb or hindlimb clonus; 4 – bilateral forelimb or hindlimb clonus; 5 – side-to-side rolling; 6 – wandering (i.e., scrambling along the monitoring chamber walls), and a bonus score of 0, 0.5, or 1 on the basis of its sub-grade (0 – mild; 0.5 – moderate; 1 – severe). The behavior score was the sum of the main and the bonus scores. Mouse activity was scored in a blind manner as the behaviors of the mice were recorded by video, and the videos were labeled by the numbers without the information of actual treatments.

### Immunofluorescence staining

The mice were sacrificed by decapitation at the first minute after the loss of righting reflex. The whole brain was harvested and used for the analysis of c-Fos immunostaining. The harvested whole brain was then fixed in 4% PFA overnight and transferred into a 15% and 30% sucrose solution until it sank to the bottom. The brain slice (14 μm) was cut by the cryostat (CM1850, Leica,  USA). After three times of washing with PBS, the slices were immersed in 10% goat serum in 0.1% PBS-0.3% Triton X for 60 minutes at room temperature. Later, the slices were incubated with rabbit monoclonal anti-c-Fos antibody (1:250, Cell Signaling, USA) at 4 °C overnight. On the second day, the brain slices were incubated with Alexa Fluor 594-conjugated donkey anti-rabbit IgG (1:500, Invitrogen, USA) for 60 minutes at room temperature. During the staining, we set the negative control group. In the negative control group, the c-Fos antibody was not added and the rest of steps were kept the same. Then, the slices were placed into a fluoroshield mounting medium with DAPI (Abcam, USA). Finally, the slices were observed under a microscope, and the images were captured by a digital camera (Keyence, Japan). During image acquisition, the exposure time was kept the same. The number of c-Fos positive neurons was accounted under the same field of 20X microscope.

### Calcium imaging *in vivo*

Genetically encoded calcium indicator GCaMP6 slow (GCaMP6s) was used for calcium imaging of L2/3 neuronal somata in the primary somatosensory cortex. GCaMP6s was expressed with the recombinant adeno-associated virus [AAV, serotype 9; >1 × 10^13^ (GC/ml) titer; produced by University of Pennsylvania Gene Therapy Program Vector Core]. AAV9-CAG-Flex-GCaMP6s and AAV9-CaMKII-Cre were mixed in a ratio of 2500:1. CaMKII promoter drives Cre expression in glutamatergic pyramidal neurons in the cortex. A total of 0.1–0.2 μL of mixed AAV was injected into L2/3 of the primary somatosensory cortex (1.3 to 1.5 mm posterior of bregma and 2.0 to 2.5 mm to the midline) using a glass microelectrode at P0. The surgical procedure for preparing awake animal imaging has been described previously^18^. In short, a head holder composed of two micro-metal bars was attached to the animal’s skull to reduce motion-induced artifacts during imaging. At P9, animals were anesthetized via an intraperitoneal injection of ketamine (100 mg/kg) and xylazine (15 mg/kg). A midline incision of the scalp exposed the periosteum, and a small skull region over the primary somatosensory cortex was located based on stereotactic coordinates and marked with ink. The skull holder was placed on top of the marked region and fixed in place using cyanoacrylate-based glue. The head holder was then further fortified with dental acrylic cement. After the dental cement was completely dry, the marked skull region of sensory cortex was thinned by a dental drill and then peeled off with fine forceps. During this process, dura over the sensory cortex was kept intact. A glass coverslip was glued to the skull, over the exposed sensory cortex. *In vivo* calcium imaging was performed in awake, head-restrained mice after the 24-hour recovery from the surgery-related anesthesia^59^. At P10, the mouse with head holder was placed on the stage of a Bruker two-photon laser scanning system equipped with a Ti:sapphire laser (Mai Tai DeepSee; Spectra Physics) tuned to 920 nm. The average laser power on the mouse brain was ~20–30 mW. All the images were taken using a 25× objective immersed in an artificial cerebrospinal fluid  solution and with a 1 × digital zoom. For each experimental point, images were collected for 30 seconds at a resolution of 512 × 512 pixels and a frame rate of 2 Hz. The analysis of imaging data was performed using NIH ImageJ software. An integrated measurement of a cell’s output activity over a 30-second recording was performed to compare neuronal activity at different time points and among different cells. *F*_0_ is the baseline fluorescence signal which is the average value over 2 seconds corresponding to the lowest fluorescence signal over the first 30-second recording period. Then Δ*F*/*F*_0_ was calculated by (*F* − *F*_0_)/*F*_0_^59^.

### Primary neuronal culture

At the 16^th^ day of gestation, pregnant mice were euthanized by carbon dioxide introduced into the chamber. Fetuses were removed by cesarean sections and transferred to the petri dish with phosphate-buffered saline (PBS). The whole brain was gently removed and placed into the dissection medium. The cortex was dissected from the intact brain, and meningeal tissues were removed carefully. The neurons were dissociated using 0.05% Trypsin with EDTA (Gibco, USA) for 15 minutes at 37 °C. The dissociated neurons were plated with neurobasal medium (Gibco) with fetal bovine serum (FBS, Gibco) in coverslips that had been precoated with poly-D-lysine (Sigma, USA). After 1 hour of incubation, the medium was changed into a neurobasal medium with B27 (Gibco). Half of the medium from each well or dish was replaced with fresh maintenance medium warmed to 37 °C every two days^60^. On the 8^th^ to the 10^th^ day after the plating, the neurons were used to perform the calcium imaging.

### Calcium imaging *in vitro*

The saturated sevoflurane stock solution was prepared in a gas-tight bottle by dissolving excess sevoflurane in the standard buffer (140 mM NaCl, 4 mM KCl, 1 mM MgCl_2_, 1.2 mM CaCl_2_, 10 mM HEPES, 5 mM D^+^ glucose, pH 7.3) overnight as described in previous studies^61^. The neurons were loaded with  1 mM Fluo4-AM (Invitrogen, USA) in a standard buffer for 30 minutes^62^. After acquiring the baseline measurement, neurons were exposed to different concentrations of sevoflurane (0.11 mM, 0.22 mM, and 0.45 mM) dissolved in the standard solution that was applied via a valve-controlled gravity-fed perfusion system with a 200-mm diameter outlet. The dye was excited at 480 ± 15 nm, and the emitted fluorescence was measured at 535 ± 25 nm. The images were captured by a digital camera (Nikon, Japan) with a 2 second interval and analyzed by using the ImageJ software. Relative fluorescence difference (Δ*F*/*F*_0_) was calculated by Δ*F*/*F*_0_ = (*F* − *F*_0_)/*F*_0_ in each image (*F*_0_ was determined as the average value before the drug application). Saturated stock solutions of sevoflurane were prepared in gas-tight bottles by dissolving excess anesthetic agents in bath solutions overnight as previously described before^61^. From these stock solutions, fresh dilutions were made up every 40–60 minutes. Concentrations of anesthetics in the bath solutions were verified by using a modified head-space gas chromatography method.

### Statistical analysis

The normality test was performed first and normal distributed data were presented as the mean ± standard error of the mean (SEM). We used a **χ**^2^ test to detect the difference in the number of mice showing hyperactivity between the control group and the anesthetic groups. The incidence of hyperactivity was calculated as the number of mice demonstrating hyperactivity. We used the Student’s *t*-test to compare the c-Fos-positive cells between the control and sevoflurane groups. A two-tailed test was used. We used ANOVA to evaluate the difference in the incidence of neuronal activity, c-Fos protein level, and calcium fluorescence among groups. *P* value < 0.05 was taken as statistically significant. All statistical analyses were performed using Prism 6 (La Jolla, USA).

## Supplementary information

Supplementary Video.

Supplementary Figures.
