# Supplementary material for: Sevoflurane induces neuronal activation and behavioral hyperactivity in young mice
Source: Sci Rep. 2020 Jul 8;10:11226. doi: 10.1038/s41598-020-66959-x (PMC7343864; doi:10.1038/s41598-020-66959-x)
Supplement: Supplementary file 2 — Supplementary Figures. [file 41598_2020_66959_MOESM2_ESM.docx]

**Supplemental Information**

**Sevoflurane induces neuronal activation and behavioral hyperactivity in young mice**

Lei Yang, Hoai Ton, Ruohe Zhao, Erez Geron, Mengzhu Li,

Yuanlin Dong, Yiying Zhang, Buwei Yu, Guang Yang and Zhongcong Xie

**Supplementary Figure 1.**


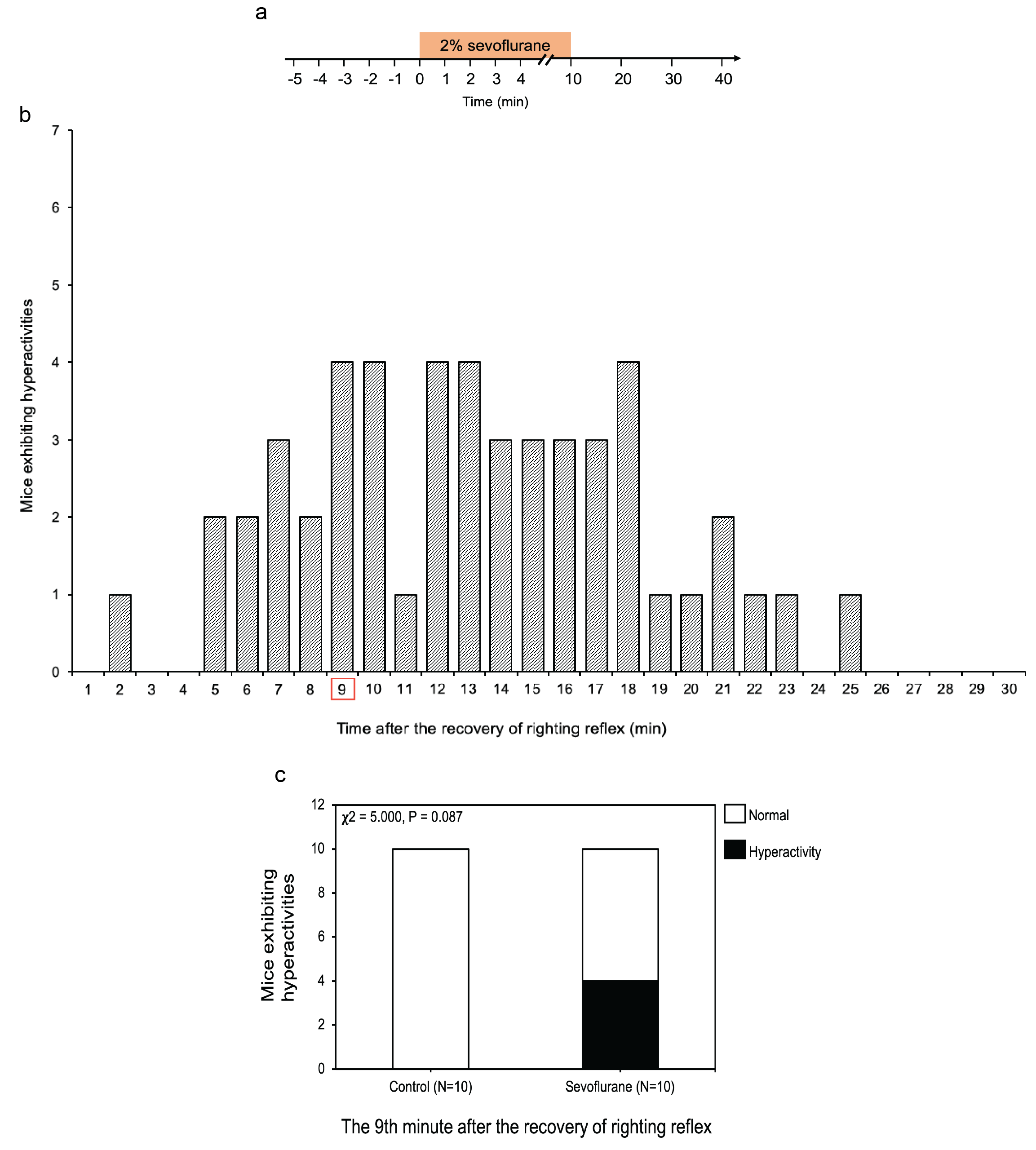


**Supplementary Fig. S1. Sevoflurane induced behavioral hyperactivity in young mice after the recovery of righting reflex. (a)** Timeline of the experiment. The activity of each of the P10 mice was recorded before, during, and after the administration of 2% sevoflurane for 10 minutes (indicated by the box). We measured the activity of the mice after the recovery of righting reflex. **(b)** The number of mice exhibiting hyperactivity after the recovery of righting reflex. **(c)** At the 9th minute after the recovery of righting reflex, 40% of young mice exhibited hyperactivity (𝛘^2^ = 5.000, *P* = 0.087, *n* = 10, 𝛘^2^ test).

**Supplementary Fig. S2**


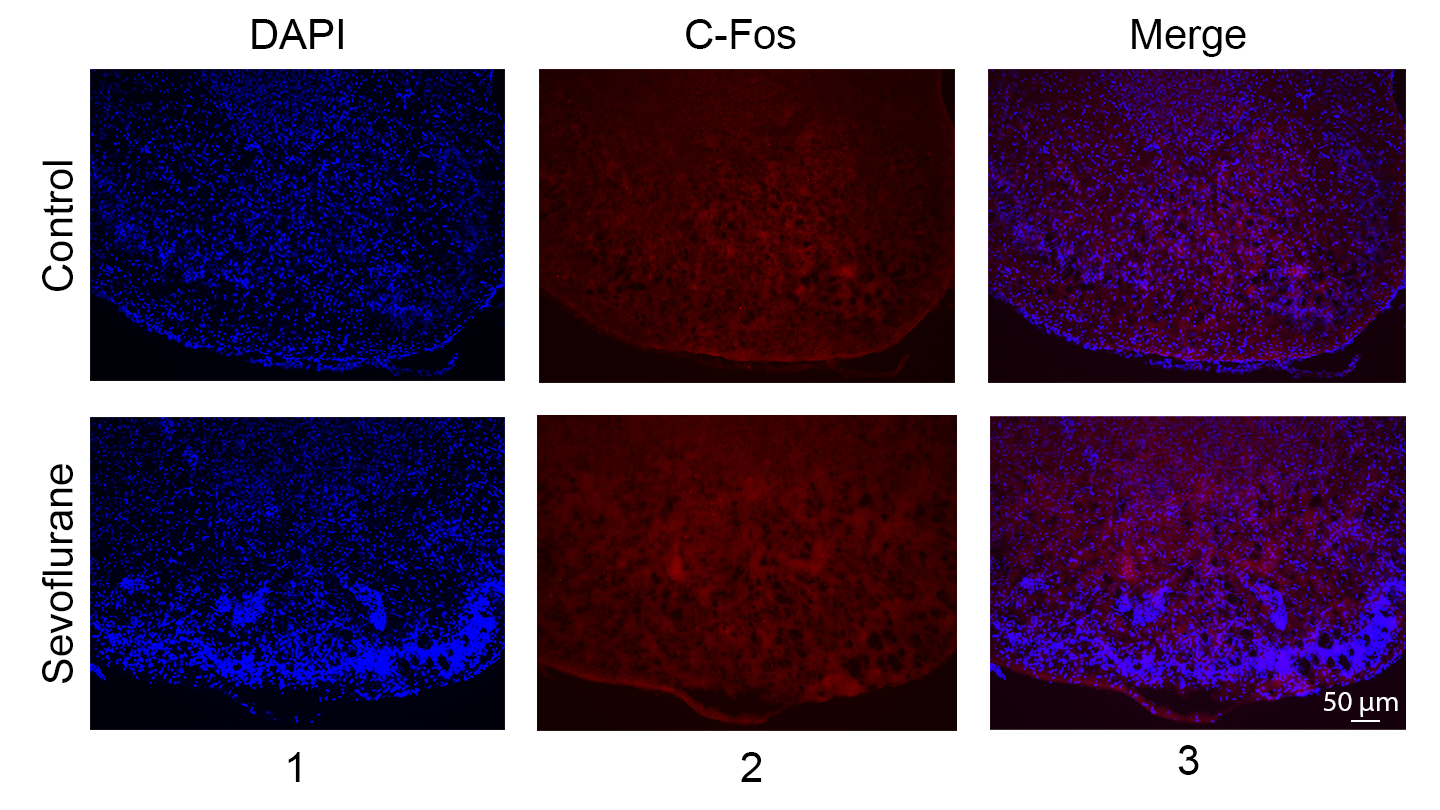


**Supplementary Fig. S2. Sevoflurane did not increase the number of c-Fos-positive cells in the olfactory cortex of young mice.** Immunohistochemistry staining of c-Fos (magnification 20 x) showed the area of the brain (olfactory cortex) with the c-Fos-positive cells (red). Column 1 is the image of nuclei (blue), column 2 is the image of c-Fos (red), and column 3 is the merged image. The top row represents the brain tissues of mice following the control condition, and the bottom row represents the brain tissues of mice treated with 2% sevoflurane. There was no significant difference in the number of c-Fos-positive cells in the olfactory cortex between the mice in the control condition group and the mice in the 2% sevoflurane group.

**Supplementary Fig. S3**

**
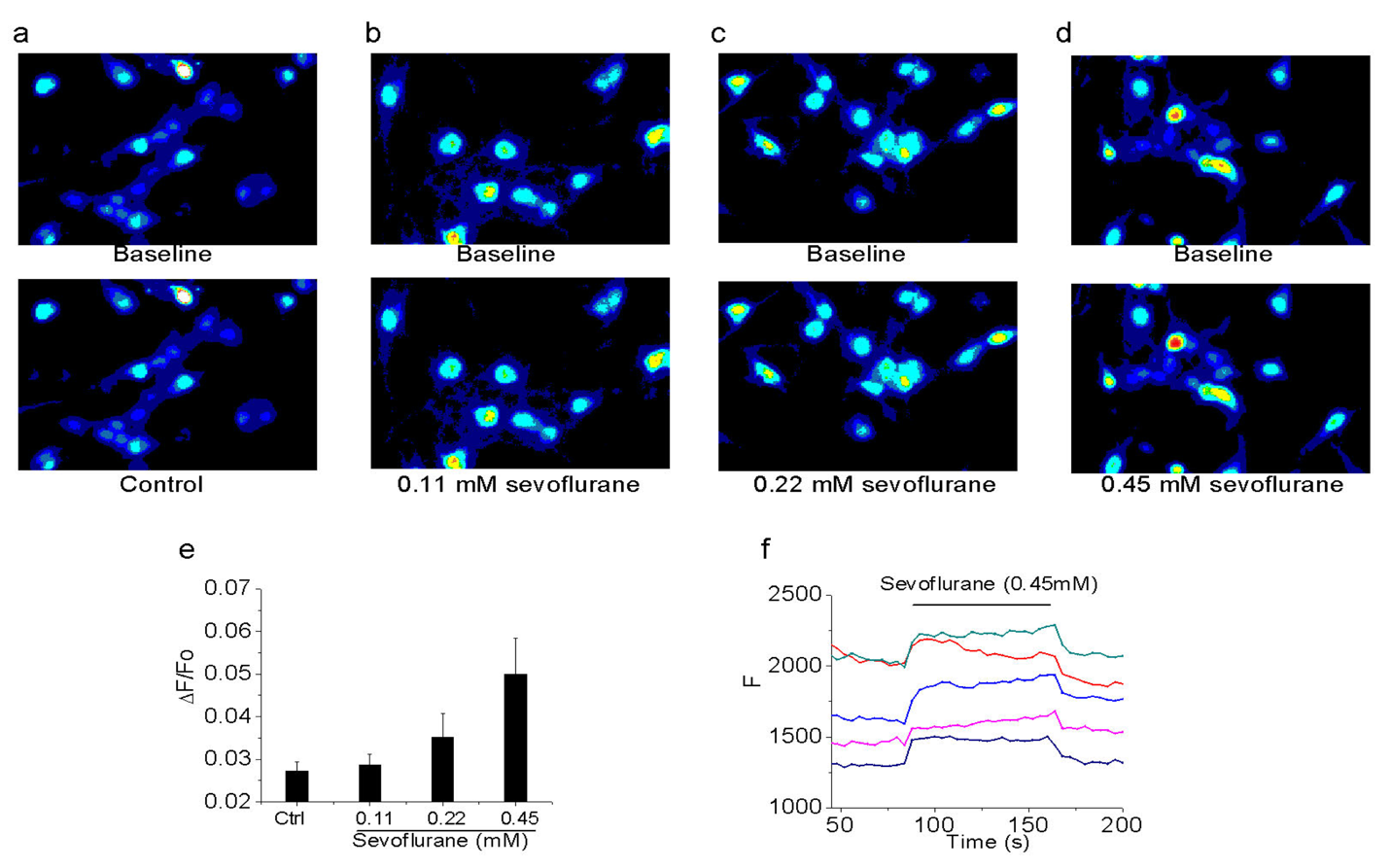
**

**Supplementary Fig. S3. Sevoflurane induced a dose-dependent increase in the intracellular calcium levels in primary neurons. (a-d)** The imaging of intracellular calcium levels in the control and the different doses of sevoflurane [0.11 mM (0.5%), 0.22 mM (1%) and 0.45 mM (2%)]. **(e)** Quantification of fluorescence showed that sevoflurane induced an increase in the intracellular calcium levels in a dose-dependent manner. In particular, the administration of 0.45 mM (2%) sevoflurane (bar 4) significantly increased the intracellular calcium concentration as compared to the control condition (*P* < 0.05, *n* = 42 cells). **(f)** Representative Fluo-4 fluorescence of neurons responded to the administration of 0.45 mM (2%) sevoflurane from 80 seconds to 160 seconds. The bar line indicated the period that cells were treated with sevoflurane. F means fluorescence intensity.

**Supplementary Fig. S4**

**
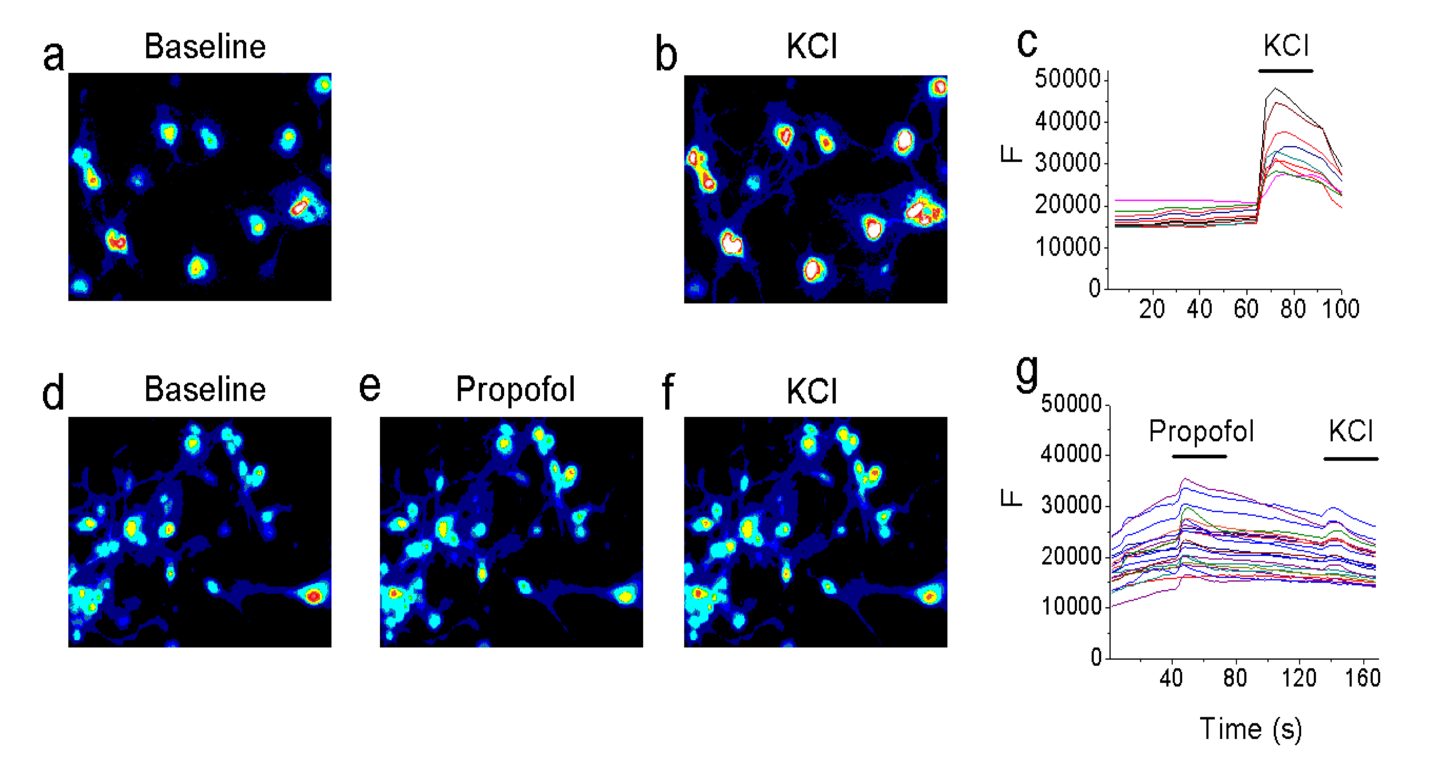
**

**Supplementary Fig. S4. Propofol attenuated the KCl-induced increase in the intracellular calcium levels. (a)** The baseline intracellular calcium imaging in the neurons. **(b)** The intracellular calcium imaging in the neurons treated by KCl (50 mM). (**c**) The quantification of the intracellular calcium imaging of a and b. (**d**) The baseline intracellular calcium imaging in the neurons. **(e)** The intracellular calcium imaging in the neurons treated with propofol (10 µM). **(f)** The intracellular calcium imaging in the neurons treated with propofol (10 µM) plus KCl (50 mM). (**g**) The quantification of the intracellular calcium imaging of d, e, and f. KCl, potassium chloride. F means fluorescence intensity.
